# Application of FLIC model to predict adverse events onset in neuroendocrine tumors treated with PRRT

**DOI:** 10.1038/s41598-021-99048-8

**Published:** 2021-09-30

**Authors:** Federica Scalorbi, Giovanni Argiroffi, Michela Baccini, Luca Gherardini, Valentina Fuoco, Natalie Prinzi, Sara Pusceddu, Enrico Matteo Garanzini, Giovanni Centonze, Margarita Kirienko, Ettore Seregni, Massimo Milione, Marco Maccauro

**Affiliations:** 1 Nuclear Medicine Department, Foundation IRCCS, Istituto Nazionale Tumori, Milan, Italy; 2 Department of Statistics, Computer Science, Applications (DiSIA), University of Florence, Florence, Italy; 3 Medical Oncology, Foundation IRCCS, Istituto Nazionale Tumori, Milan, Italy; 4 Diagnostic Imaging Department, Foundation IRCCS, Istituto Nazionale Tumori, Milan, Italy; 5 Pathology Unit, Foundation IRCCS, Istituto Nazionale Tumori, Milan, Italy

**Keywords:** Cancer, Endocrinology, Gastroenterology, Medical research

## Abstract

To develop predictive models of side effect occurrence in GEPNET treated with PRRT. Metastatic GEPNETs patients treated in our centre with PRRT (177Lu-Oxodotreotide) from 2019 to 2020 were considered. Haematological, liver and renal toxicities were collected and graded according to CTCAE v5. Patients were grouped according with ECOG-PS, number of metastatic sites, previous treatment lines and therapies received before PRRT. A FLIC model with backward selection was used to detect the most relevant predictors. A subsampling approach was implemented to assess variable selection stability and model performance. Sixty-seven patients (31 males, 36 females, mean age 63) treated with PRRT were considered and followed up for 30 weeks from the beginning of the therapy. They were treated with PRRT as third or further lines in 34.3% of cases. All the patients showed at least one G1–G2, meanwhile G3–G5 were rare events. No renal G3–G4 were reported. Line of PRRT administration, age, gender and ECOG-PS were the main predictors of haematological, liver and renal CTCAE. The model performance, expressed by AUC, was > 65% for anaemia, creatinine and eGFR. The application of FLIC model can be useful to improve GEPNET decision-making, allowing clinicians to identify the better therapeutic sequence to avoid PRRT-related adverse events, on the basis of patient characteristics and previous treatment lines.

## Introduction

Neuroendocrine tumors (NETs) are a heterogeneous group of well differentiated neoplasms, considered rare malignancies, even if the incidence has increased more than 6 times in the last decades^1^. Gastroenteropancreatic Neuroendocrine Tumors (GEPNET) constitutes a subgroup of NETs which are diagnosed as low-grade lesions in the majority of cases. The Surveillance, Epidemiology End Results (SEER) database has estimated an incidence of 3.56/100,000 per year in the USA. The incidence in Europe appears to be lower and ranges between 1.33 and 2.33/100,000 per year, although the data are retrospective and heterogeneous^2–4^. According to WHO 2019 classification^5^, the GEPNETs are classified into three groups, on the basis of the proliferation index (Ki-67) and the number of mitotic figures per 2 mm^2^. Ki-67 < 3% and/or mitotic index < 2 HPF (high power fields) are classified as G1, Ki-67 3–20% and/or mitotic index 2–20 HPF as G2 and Ki-67 > 20% and/or mitotic index > 20 HPF as G3.

Although GEPNET are considered low-growing tumors, up to 90% of patients have lymph node metastasis and in 45–70% of cases liver metastasis are present at the time of diagnosis^6,7^. Surgery of primary tumor represents the only curative treatment, with a 5-year survival rate between 60–80%. In case of liver metastasis, only 5–15% of patients are eligible for liver metastasIs resection^7,8^*.* At the present, Somatostatin Analogues (SSA) represents the first treatment in the management of unresectable disease, reducing both hormone production and tumor growth. In case of progression, even after SSA dosage increase, local or systemic approaches have to be considered. The burden of disease, the location of primary tumor and metastasis as well as the tumor biology are the main aspects to consider in the treatment choice. For oligometastatic liver disease, a loco-regional approach can be applied as ablation or trans-arterial chemoembolisation (TACE) or radioembolisation (TARE). In case of progressive metastatic spread, a systemic approach is required and must be selected in accordance with the primary tumor localisation. For midgut patients, PRRT and everolimus (an mTOR inhibitor) are the two main choices^9,10^. For foregut patients the choices are multiple, starting from molecular targeting therapies (everolimus and Sunitinib (a tyrosine kinase inhibitor))^11^, following with alkylating chemotherapies as Capecitabine and Temozolomide combination^12^ to end with PRRT^13–18^.

Since metastatic GEPNET are low-growing neoplasms, characterised by stability followed by progression, multiple lines of treatment have to be adopted to control distant localisations and symptoms. Hence, therapy-related adverse events can occur during the course of the disease and can forbid further therapeutic lines. Currently, there are some guidelines and flow chart^19^ to support decision making in order to assess the best treatment choice according to disease burden, somastostatin receptor expression and previous treatment protocols. Nevertheless, each case features specific conditions and frequently requires a custom evaluation. In this scenario, there are no precise models to assess the occurrence of adverse events during PRRT protocol in GEPNET patients treated with previous lines of systemic therapies. The ^177^ Lu-DOTATATE PRRT, a recently approved target therapy (EMA, AIFA)^20,21^, can be applied in second or further line in well differentiated disease, after SSA failure. Actually, the sequence and timing of treatments are frequently related to single-centre experience, in particular in case of foregut NET for which no specific guidelines are available. For midgut patients in the majority of cases PRRT is administered as second line, after SSA failure. The variability in PRRT treatment line can deeply impact on therapy effectiveness, being a real cause of PRRT discontinuation related to haematological, hepatic or renal toxicities. To nowadays, there are only some retrospective publications regarding the evaluation of PRRT adverse events in correlation with previous line of treatments, showing associations between treatment choice and toxicities development^22–26^. *Patient’s baseline characteristics are possible predictors of toxicity development as well, but their role is scarcely explored in the literature*^27^.

The aim of this study is to investigate the occurrence of adverse events in GEPNET patients during radio-metabolic treatment and to evaluate the role of previous therapies and individual characteristics in predicting treatment-related toxicities. Considering that G3-G4 toxicities are rare events during PRRT treatment, issues related to perfect prediction occurred in the analysis of our dataset (data separation). Therefore, we adopted a penalised regression approach based on the Firth’s logistic regression with intercept correction (FLIC model) to solve this problem^28^. A backward selection procedure was implemented to detect the most relevant predictors.

## Patients and methods

### Data collection

We collected data from a cohort of 87 consecutive patients with G1-G2 GEPNETs, scheduled to receive PRRT through the Foundation IRCCS, Istituto Nazionale Tumori, Milan, from April 2019 to December 2020. All the patients were treated with ^177^Lu-Oxodotreotide (Lutathera®), 7.4 GBq iv per administration, interval 8 weeks (+/− 2 weeks), following EMA and AIFA indications^20,21^. Patients were treated with 1 to 4 PRRT administrations and were all previously treated with SSA, followed by disease progression confirmed by CT or MRI scan. The analysis was performed on the subset of patients enrolled from April 2019 to June 2020 (n = 67), following up them for 30 weeks from the first PRRT administration. Before PRRT, tumor burden and somastostatin receptor expression were evaluated by CT/MRI scan and 68 Ga-DOTA-SSA PET/Octreoscan, respectively. Inclusion criteria were: age ≥ 18 years, histopathological diagnosis of well differentiated G1 or G2 (WHO 2019) GEPNET, at least one previous line of treatments with SSA and a CT/MRI scan performed before PRRT, repeated after 2 administrations. Haematopoietic, liver and renal functions before the first PRRT administration were considered as reference baseline. Hematochemical tests were re-evaluated every 14 days during the whole treatment; haematological, renal and liver toxicities were graded applying Common Terminology Criteria for Adverse Events version 5.0 (CTCAE) and the nadir values were reported per patient. Haematopoietic toxicities were evaluated considering anaemia, white blood cells (WBC), neutrophils and platelets counts. ALT/GPT (alanine aminotransferase), AST/GOT (aspartate aminotransferase), GGT (gamma glutamyl transpeptidase), total bilirubin, albuminaemia and INR values were considered to evaluate liver function alteration. To assess renal function, creatinine clearance and eGFR values were considered. Since eGFR can be correctly assessed only in patients younger than 75 years, older patients have been excluded from the statistical analysis of this outcome. Exclusion criteria were: age < 18 years, absence of primary tumors diagnosis, absence of radiological and nuclear medicine imaging evaluation (baseline and interim), absence of signed informed consent.

The Performance Status was assessed by ECOG score, before PRRT treatment. The study population was subdivided as midgut/foregut in relation to primary tumor localisation and as G1 or G2 in accordance with WHO 2019 classification (data were extracted from pathological reports). In doubtful cases, a pathological evaluation of paraffin samples was required. The site of secondary localisations as well as the number of metastatic organs involved were evaluated per patient. Furthermore, previous lines of treatment were evaluated and patients were grouped as follows: PRRT as second or as third/further line of treatment. The following previous therapies were also considered as possible predictive factors of CTCAE onset: splenectomy, everolimus, alkylating chemotherapy and MetNET protocol (Lanreotide and Metformin)^29^. Surgical resection of primary tumor, loco-regional therapies and liver transplantation were not included in the lines of treatment, nor SSA shift or SSA increase dosage.

### Ethical approval

This observational study was approved by the Ethical Committee of National Institute for Tumor of Milan (Study number: INT 6/20) and was conducted in accordance with the Principles of Declaration of Helsinki (1964). All the patients have given written informed consent for the participation in the study.

### Statistical analysis

The collected variables were summarized as mean (± standard deviation) when continuous and as frequency and percentage (%) when categorical. The crude associations between occurrence of adverse events and patient characteristics, including previous treatments, were evaluated by Fisher’s test and Chi square test. Age differences between groups were evaluated by Wilcoxon’s test.

With the aim of investigating the association between the occurrence of each specific adverse event and the covariates (primary localisation, WHO grading, patient characteristics, treatment lines, number of previous therapies, number of secondary localisations), a FLIC model was specified. The FLIC model modifies the more known Firth’s logistic regression^30^, which is widely used to overcome separation problem frequently arising when, as in our application, the logistic regression is applied to relatively small datasets, with rare events, characterized by unbalanced distribution of the risk factors between outcome categories and high correlation between explanatory variables. The FLIC model introduces a post-hoc adjustment of the intercept of the Firth’s logistic regression providing unbiased predictive probabilities. A backward procedure for variable selection was used to detect the patient’s characteristics that were more predictive of the outcomes^31^.

The predictive performances of the procedure and the stability of the variable selection were assessed by using a subsampling approach^32^. In particular, we generated 100 subsamples from the original dataset by repeatedly sampling 63.2% of the subjects, with no repetitions. Then we performed on each subsample the previously described analysis, obtaining 100 different backward selection results. We calculated for each covariate the frequency of selection and the mean absolute effect over the 100 repetitions. Finally, the Area Under the ROC Curve (AUC) was calculated for each selected model on 36.8% of patients not included in the subsample. The overall predictive performance was calculated as average AUC over the 100 repetitions.

STATA v16.0 was used for data management and descriptive analyses. The analysis based on FLIC logistic regression has been performed by using the package *logistf* of R software^33,34^.

## Results

In Table 1 we described the cohort of 67 patients (31 (46.3%) males, 36 (53.7%) females, mean age 63 years, SD ± 11, 95% CI 60.4–65.6) included in the analysis. According to the primary localisation and WHO 2019 classification, 38 (56.7%) patients were classified as midgut, 29 (43.3%) as foregut meanwhile 24 (35.8) as G1 and 43 (64.2) as G2. Evaluating ECOG performance status before PRRT, 55 (82.1%) patients were classified as ECOG 0 and 12 (17.9%) as ECOG 1 or 2. Metastasis were diagnosed in all the patients and, in the majority of cases, were located in the liver (66, 98.5%), followed by lymph nodes (37, 56.1%) and bone (23, 34.8%) (Table 1). Sixty-one (91%) patients performed all the 4 PRRT administrations, 64 (95.5%) at least three and 66 (98.5%) at least two. Of the six patients who did not complete the entire cycle of radiometabolic treatment, two interrupted it due to toxicity occurrence, the remaining four due to clinical/radiological progression. One of these four patients died for clinical progression not related to toxicity after 26 weeks from the first PRRT administration. The majority of patients were treated with PRRT as second line (45, 67.2%); 21 with PRRT as third or further line (32.8%). The great majority of midgut patients were treated with PRRT as second line (30, 78.9%) meanwhile PRRT was administered as second line in just half of the foregut population (15, 51.8%), followed by third and further lines in a quarter (7, 24.1%). Table 1 shows the distribution of the previous lines of treatment. All the patients have been previously treated with SSA whereas 49 (73.1%) were also subjected to resection of primary tumor and 11 (16.4%) to splenectomy. Alkylating chemotherapy and everolimus were the previous treatments in 13 (19.4%) patients, in both cases.

**Table 1:** Selected study population.

**Age**	*n* = 67
Mean (± SD)	63 (± 11)
> 60 ys	38 (56.7)
**Gender**	*n (%)*
Male	31 (46.3)
Female	36 (53.7)
**ECOG PS**	
0	55 (82.1)
1_2	12 (17.9)
**Grading (WHO 2017)**	
G1	24 (35.8)
G2	43 (64.2)
**Primary tumor**	
Midgut	38 (56.7)
Foregut	29 (43.3)
**Distant metastasis localisation**	
Liver	66 (98.5%)
Nodes	37 (56.1)
Bone	23 (34.8)
Mesentery	11 (16.7)
Peritoneum	7 (10.6)
Lung	1 (1.5)
Other localisations	7 (10.6)
**PRRT administrations**	
I	67 (100I)
II	66 (98.5)
III	64 (95.5)
IV	61 (91)
**PRRT line of treatment**	
2nd	45 (67.2)
*Midgut*	30 (78.9)*
*Foregut*	15 (51.8)*
3rd	13 (19.4)
*Midgut*	6 (15.8)*
*Foregut*	7 (24.1)*
4th or further	9 (13.4)
*Midgut*	2 (5.3)*
*Foregut*	7 (24.1)*
**Previous therapies**	
Surgery	49 (73.1)
Loco-regional (TACE, TARE, RT)	12 (17.9)
Splenectomy	11 (16.4)
Alkylating chemotherapy	13 (19.4)
mTOR inhibitor (Everolimus)	13 (19.4)
MetNET protocol	5 (7.5)

TACE, transarterial chemoembolisation; TARE, transarterial radioembolisation. *percentage calculated considering midgut and foregut separately.

Table 2 illustrates the occurred toxicities and highlights that G3–G4 CTCAE were extremely rare events, reported in 5 (7.5%) cases considering haematological alterations (2 neutropaenia, 1 anaemia, 2 thrombocytopaenia) and 2 (3%) cases for liver alteration (1 ALT and 1 INR). Anaemia and thrombocytopaenia occurred in the same patient were the cause of PRRT discontinuation, after the third administrations, and required medical procedures to restore normal values. In all the other cases the G3–G4 alterations were transitional events and, therefore, PRRT protocol was completed. No G3–G4 renal toxicities were reported. All the patients showed at least one G1-G2 CTCAE during PRRT protocol, in particular eGFR alteration (44, 75.9%), followed by anaemia (46, 68.6%), thrombocytopenia (32, 47.8%) and leukopenia (30, 44.8%). Since eGFR was assessed in 58 patients only, in accordance with age cut-off, the percentage of G1–G2 resulted particularly high although the absolute number of cases was comparable with anaemia occurrence (44 versus 46, see also Table 2).

**Table 2:** Occurred CTCAE. *Percentages calculated in accordance with the number of patients (n = 67).

Adverse events	G1-G2, n(%)*	G3-G4, *n*(%)	Total CTCAE, n(%)
**Leukopaenia**	**30 (44.8)**	–	**30 (44.8)**
*midgut*	19 (63.3)	–	19 (63.3)
*foregut*	11 (36.7)	–	11 (36.7)
**Neutropaenia**	16 (23.9)	2 (3)	18 (26.9)
*midgut*	9 (56.3)	1 (50)	10 (55.6)
*foregut*	7 (43.7)	1 (50)	8 (44.4)
**Anaemia**	**46 (68.6)**	1 (1.5)	**47 (70.1)**
*midgut*	26 (56.5)	1 (100)	27 (57.5)
*foregut*	20 (43.5)	–	20 (42.5)
**Thrombocytopaenia**	**32 (47.8)**	2 (3)	**34 (50.7)**
*midgut*	21 (65.6)	1 (2.6)	22 (64.7)
*foregut*	10 (31.3)	1 (3.5)	11 (32.4)
**ALT/GPT increase**	21 (31.3)	1 (1.5)	22 (32.8)
*midgut*	10 (47.6)	–	10 (45.4)
*foregut*	11 (52.4)	1 (100)	12 (54.5)
**AST/GOT increase**	17 (25.4)	–	17 (25.4)
*midgut*	8 (47.1)	–	8 (47.1)
*foregut*	9 (52.9)	–	9 (52.9)
**GGT increase**	12 (17.9)	–	12 (17.9)
*midgut*	3 (25)	–	3 (25)
*foregut*	9 (75)	–	9 (75)
**Total bilirubin increase**	17 (25.4)	–	17 (25.4)
*midgut*	8 (47.1)	–	8 (47.1)
*foregut*	9 (52.9)	–	9 (52.9)
**Albumine decrease**	4 (6)	–	4 (6)
*midgut*	2 (50)	–	2 (50)
*foregut*	2 (50)	–	2 (50)
**INR increase**	6 (9)	1 (1.5)	7 (10.5)
*midgut*	2 (33.3)	1 (2.6)	3 (42.9)
*foregut*	4 (66.6)	–	4 (57.1)
**Creatinine clearance**	16 (23.9)	–	16 (23.9)
*midgut*	10 (62.5)	–	10 (62.5)
*foregut*	6 (37.5)	–	6 (37.5)
eGFR decrease(n:58)**	**44 (75.9)**	–	**44 (75.9)**
*midgut (n: 31)*	21 (47.7)	–	21 (47.7)
*foregut (n: 27)*	23 (52.3)	–	23 (52.3)

The numbers that are highlighted in bold are referred to the most frequent reported adverse events.

**eGFR alteration was assessed in 58 patients, in accordance with age cut-off.

The marginal association between the reported CTCAE, patient’s characteristics, the number of previous treatment lines, the previous alkylating chemotherapy, everolimus, MetNET and splenectomy was assessed. Pearson’s chi square, Fisher’s and Wilcoxon’s test were applied to the frequencies of events. Relevant associations were observed for: aging and eGFR increase, female gender and anaemia, ECOG-PS 1–2 and increase of creatinine level, line of PRRT administration and increased GGT level. Splenectomy was inversely associated with thrombocytopenia and ALT/GPT increase. Table 3 shows in detail the results and the correspondent p values. No crude associations were reported between previous treatment with everolimus or chemotherapy and CTCAE occurrence.

**Table 3:** P-values of Chi square/Fisher tests to evaluate the association between CTCAE and the independent variables; p-value of Wilcoxon’s test for variable Age.

Explanatory variables	Leukopaenia	Neutropaenia	Anaemia	Thr-paenia	ALT increase	AST increase	GGT increase	Bil increase	Alb decrease	INR increase	Creatinine increase	eGFR decrease
Age	0.589	0.547	0.255	0.757	0.572	0.483	0.775	0.758	0.159	0.875	0.436	**0.032**
Gender	0.219	0.583	**0.003**	0.028	0.298	1	0.524	0.269	0.329	0.696	0.011	0.378
ECOG-PS	1	0.720	0.091	0.539	1	0.715	0.678	1	0.144	0.6	**0.029**	0.322
Grading	1	0.567	0.405	0.131	1	1	1	0.574	1	0.407	1	0.118
Liver	1	0.069	0.511	1	0.538	1	0.328	1	1	1	0.423	0.428
Nodal	1	0.592	1	0.627	0.792	0.386	0.755	0.168	**0.036**	1	0.574	1
Mesenteric	0.199	1	0.282	0.34	0.48	0.053	1	1	1	0.323	0.438	0.673
Peritoneal	0.692	0.375	0.094	0.259	0.416	1	1		0.669	1	1	1
Bone	0.608	0.773	0.402	1	0.005	0.547	1	0.56	0.113	0.221	0.77	0.338
Lung	0.448	1	1	0.493	1	1	1	1	1	1	1	1
PRRT line	0.082	0.166	0.102	0.504	0.718	0.116	**0.047**	0.645	0.398	0.062	0.628	0.714
Chemotherapy	0.759	0.312	0.315	0.765	0.499	0.715	0.228	0.725	1	0.127	1	0.424
Everolimus	0.759	0.736	0.74	0.369	1	0.465	1	0.158	0.167	0.614	0.274	0.691
MetNET	0.650	0.116	1	0.197	0.316	0.588	0.216	0.099	1	0.081	1	0.563
Splenectomy	**0.017**	0.714	1	**0.006**	**0.024**	1	0.099	1	1	1	0.274	0.424

bold values indicate statistical significance, in accordance with the conventionally accepted threshold (< 0.05).

Thr-paenia: thrombocytopaenia.

**Figure 1:**
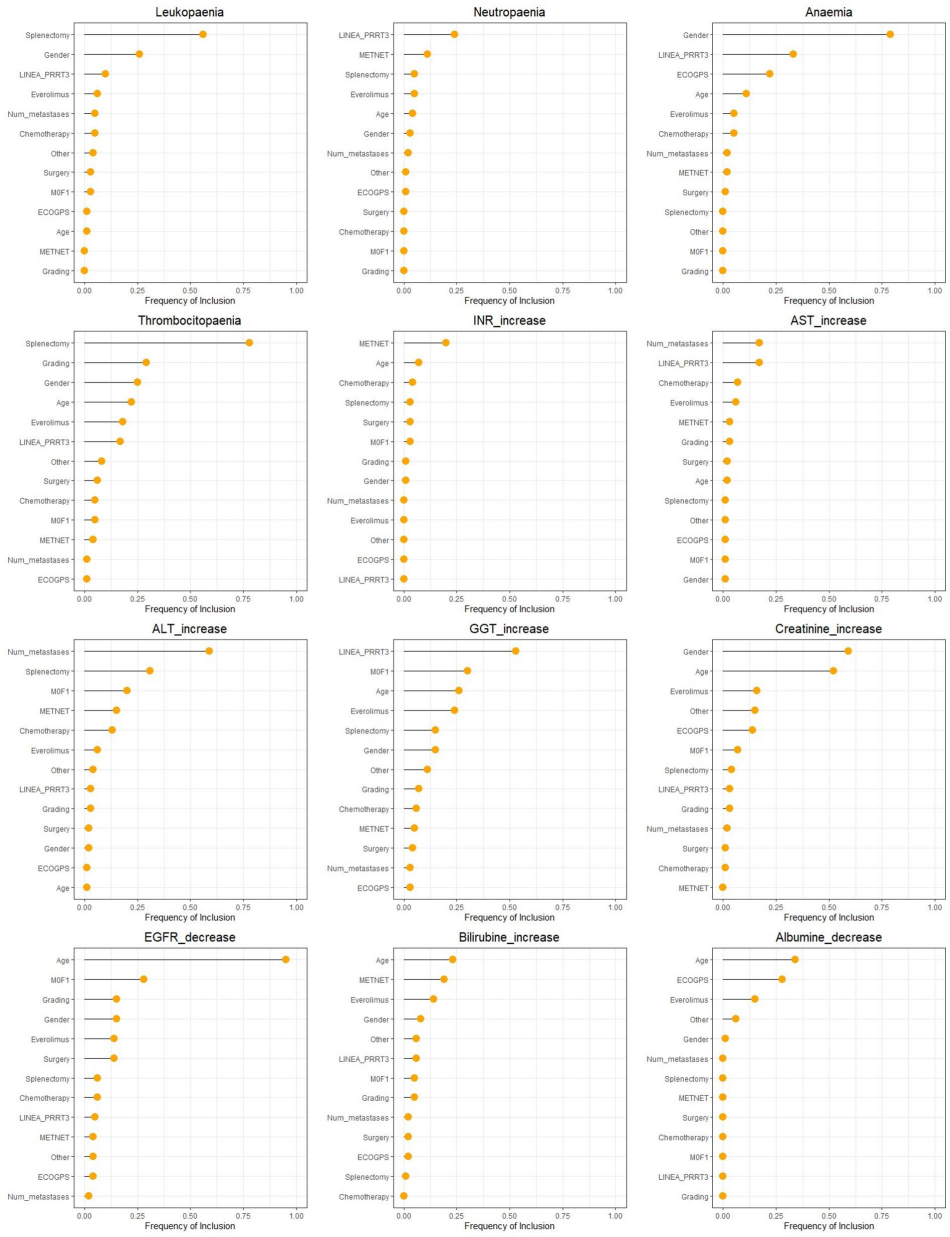
Bootstrap results: frequency of inclusion of the covariates in the FLIC model, by outcome. Grading: WHO grading.

Performing standard logistic regression analysis on the adverse event outcomes of interest, we obtained extremely high OR estimates for many covariates, with huge standard errors and wide confidence intervals, for the effect of data separation (data not reported). As shown in Table 4, under FLIC model these phenomena are partly, even if not completely, reduced. Line of PRRT administration is selected as predictor of anaemia (logOR 5.63, PRRT in second line as reference category), thrombocytopaenia (logOR 1.54), neutropaenia (logOR 0.99), GGT increase (logOR 1.88) and albuminaemia decrease (logOR < 0.01). However, the inclusion frequency (obtained from subsampling procedure) are quite low (see Fig. 1). Gender is a predictor of anaemia (logOR 1.98, male as reference category), leukopaenia (log OR 1.02), thrombocytopenia (logOR -1.08) and creatinine increase (logOR – 1.84), with frequency of inclusion larger than 60% for anaemia and creatinine. Age, modelled as continuous variable, is selected as predictor of thrombocytopenia (logOR 0.05), creatinine (logOR,0.08), eGFR (logOR 0.14), bilirubin (logOR 0.05) and albumine (logOR -0.61) with frequencies of inclusion > 50% only for creatinine and eGFR. Splenectomy is a protective factor of thrombocytopenia (logOR -2.9) and leukopaenia (logOR -2.32), with high inclusion frequencies, and of ALT increase (logOR -2.25) with an inclusion frequency < 30% (Fig. 1).

**Table 4:** FLIC logistic regression.

Outcome	Covariates	Comparison	OR	Log OR	Lower CI	Upper CI
*Anaemia*	*PRRT Line*	> *2 vs* = *2 (ref)*	279.82	5.63	− 0.39	11.66
	*ECOG-PS*	*1–2 vs 0 (ref)*	17.13	2.84	− 0.12	5.80
	*Gender*	*female vs male(ref)*	7.26	1.98	0.71	3.25
	*MetNET*	*yes vs no (ref)*	0.02	− 3.80	− 8.93	1.32
	*Everolimus*	*yes vs no (ref)*	0.01	− 4.90	− 10.78	0.97
*Thrombocyto-paenia*	*PRRT Line*	> *2 vs* = *2 (ref)*	4.66	1.54	0.14	2.94
	*Age*	*(continuous)*	1.05	0.05	− 0.01	0.11
	*Gender*	*female vs male (ref)*	0.34	− 1.08	− 2.23	0.08
	*Grading WHO2019*	*G2 vs G1 (ref)*	0.29	− 1.24	− 2.49	0.01
	*Splenectomy*	*yes vs no (ref)*	0.06	− 2.90	− 5.29	− 0.51
*Leukopaenia*	*Gender*	*female vs male(ref)*	2.76	1.02	− 0.04	2.08
	*Splenectomy*	*yes vs no (ref)*	0.10	− 2.32	− 4.23	− 0.40
*Neutropaenia*	*PRRT Line*	> *2 vs* = *2 (ref)*	2.70	0.99	− 0.12	2.11
*INR_increase*	*MetNET*	*yes vs no (ref)*	7.47	2.01	0.03	4.00
*AST-increase*	*(null model)*					
*ALT-increase*	*Midgut- Foregut*	*foregut vs midgut (ref)*	3.44	1.24	0.00	2.47
	*N° of metastasis*	*(continuous)*	0.51	− 0.68	− 1.35	− 0.01
	*Splenectomy*	*yes vs no (ref)*	0.11	− 2.25	− 4.32	− 0.18
*GGT-increase*	*PRRT Line*	> *2 vs* = *2 (ref)*	6.56	1.88	0.22	3.54
	*Midgut- Foregut*	*foregut vs midgut (ref)*	3.81	1.34	− 0.09	2.77
	*Everolimus*	*yes vs no (ref)*	0.17	− 1.79	− 3.79	0.22
*Creatinine-increase*	*Everolimus*	*yes vs no (ref)*	19.59	2.98	− 0.45	6.40
	*Age*	*(continuous)*	1.08	0.08	0.02	0.15
	*Gender*	*female vs male(ref)*	0.16	− 1.84	− 3.22	− 0.47
	*PRRT Line*	> *2 vs* = *2 (ref)*	0.12	− 2.15	− 5.38	1.07
*eGFR-decrease*	*Grading WHO2019*	*G2 vs G1 (ref)*	4.14	1.42	− 0.06	2.90
	*Age*	*continuous variable*	1.15	0.14	0.06	0.22
*Bilirubin-increase*	*MetNET*	*yes vs no (ref)*	4.77	1.56	− 0.38	3.51
	*Age*	*(continuous)*	1.05	0.05	− 0.01	0.11
	*Everolimus*	*yes vs no (ref)*	0.21	− 1.54	− 3.53	0.45
*Albumine-increase*	*ECOG-PS*	*1–2 vs 0 (ref)*	#	18.66	1.21	36.11
	*Everolimus*	*yes vs no (ref)*	#	16.64	0.41	32.87
	*Midgut- Foregut*	*foregut vs midgut (ref)*	17.08	2.84	− 1.03	6.71
	*Age*	*(continuous)*	0.55	− 0.61	− 1.18	− 0.03
	*N° of metastasis*	*(continuous)*	0.04	− 3.15	− 6.32	0.02
	*PRRT Line*	> *2 vs* = *2 (ref)*	0.00	− 14.19	− 28.33	− 0.04

Estimated associations are expressed as OR and logOR. #: hyper-inflated estimates, not reported.

Unexpectedly, chemotherapy was not a predictive factor of CTCAE onset. Everolimus was found to be a strong predictor (logOR 2.98) of creatinine increase but the inclusion frequency resulted low (Table 4 and Fig. 1). Renal toxicity was also evaluated by eGFR decrease, the predictor of which was WHO grading (logOR 1.42) but the inclusion frequency resulted low. The FLIC penalisation appears to have a poor effect in reducing separation on the albumin model, with huge coefficients for everolimus and ECOG performance status (Table 4).

Table 5 reports the average AUC, calculated for each outcome. The predictive performance of the model is above 65% for the following outcomes: anaemia, creatinine and eGFR. The mean absolute effects of the covariates arisen from subsampling are reported in the Tab S1.

**Table 5:** Average AUC obtained from subsampling, by outcome.

Outcomes	Mean AUC
Leukopaenia	0.5574
Neutropaenia	0.4709
Anaemia	**0.6739**
Thrombocytopaenia	0.6199
INR_increase	0.4286
AST_increase	0.4652
ALT_increase	0.5941
GGT_increase	0.5485
Bil_increase	0.4876
Creatinine_increase	**0.6503**
eGFR_decrease	**0.7282**
Albumine_decrease	0.5331

The outcomes that have a predictive performance above 65% are highlighted in bold.

## Discussion

The safety of PRRT treatment has been confirmed by many studies, starting with the Rotterdam experience of Kwekkeboom^35^, moving to the NETTER-1 published by Strosberg^9^, ending with the recent paper of Chen regarding an elderly cohort^1^. In comparison with these studies, we have reported a similar proportion of G3-G4 CTCAE (8.96%, only 6 patients on 67 showed severe adverse events). As expected, bone marrow toxicities were the most common G3-G4 events (5 cases on 7, 71.4%), if compared to renal and liver ones. In our study we have reported no G3-G4 renal toxicities, in accordance with NETTER-1, Chen and Fross-Baron studies^1,9,36^. We found that there is a marginal association between splenectomy and the risk of leukopaenia and thrombocytopaenia (Table 3); this highlights how surgical spleen removal is a protective factor of the reported haematological toxicity. In a recent study Medaer et al. observed significantly lower level of leukopaenia, lymphopaenia and thrombocytopaenia in the cohort pre-treated with splenectomy^37^.

Line of PRRT administration resulted to be frequently selected as a predictor of CTCAE. In particular, patients treated with PRRT as third or further line have a higher probability to develop, during radio-metabolic treatment, anaemia, thrombocytopaenia, neutropaenia, GGT increase and albuminaemia decrease. However, on the basis of the subsampling procedure, the frequencies of inclusion of PRRT line in the models were quite low, with the exception of the one calculated for the GGT increase model. Therefore, if our results are suggestive of a possible role of line of PRRT in the occurrence of CTCAE, on the other hand our conclusions regarding this predictor are not particularly strong. We would like to point out that, to nowadays, there are no studies that have correctly assessed line of PRRT as a predictive factor of CTCAE onset.

In our analysis no increased risks of CTCAE have been found in patients pre-treated with alkylating chemotherapy (Tables 3, 4). Similar results have been reported by Bergsma et al. in 2015 and Rudisile in 2019^22,38^. To note, in our study, alkylating chemotherapy was the previous treatment in 19.4% of patients (Table 1b), a percentage that is comparable to the one reported by Rudisile (20%) but not to Bergsma (12%). Fross-Baron evaluated PRRT safety in pancreatic NET heavily pre-treated with chemotherapy, showing that bone marrow toxicity was unrelated to type and length of chemotherapy. Conversely, Chen et al. reported an increased trend of G3-G4 CTCAE onset in patients who received chemotherapy before PRRT, without reaching statistical significance^1^.

In line with the literature, our results showed that previous treatment with everolimus is not a strong predictor of adverse events occurrence during PRRT (Table 4). Indeed, even if everolimus has been selected in some models as predictor, its frequencies of inclusion were always low (Fig. 1). Medaer et al.^37^ have shown no significant differences in the number of haematological CTCAE reported during PRRT between patients pre-treated with everolimus and/or Sunitinib and patients not pre-treated with target therapies. To note, neither renal nor liver toxicity were assessed in the study. Panzuto et al.^23^ have highlighted how patients previously treated with PRRT and chemotherapy have a 12-fold increased risk of severe toxicity (G3–G4) when treated with everolimus, compared to 3.68-fold for chemotherapy only and 2.58 for PRRT only.

In our study, the major predictor of liver function alteration, expressed as GGT increase, is the line of PPRT administration. The frequency of inclusion of this covariate in the GGT predictive model is quite high, indicating good reproducibility of this result. Fatal liver toxicity was reported in a single case by Fross-Baron meanwhile Chen et al. reported a G3/G4 case of ALP/GGT increase, correspondent to 1.4% of all the G3/G4 reported events^1^. Furthermore, in our study, the localisation of primary tumor (midgut versus foregut) resulted a predictor of INR increase and albumine decrease but with quite low frequencies of inclusions. The strongest predictors of renal toxicity were gender (with higher risk for males) and age (increasing risk with age), with good frequency of inclusion in the model.

Since G3-G4 are rare occurrence, classical logistic regression is not the most effective approach to be used in particular in small datasets. Indeed, in these settings, extreme regression coefficient estimates and very wide confidence intervals can be obtained. The Firth’s model has been successfully applied in different clinical fields, for example to predict major bleeding among patients using anticoagulants, to identify rare anaesthesia-related risk factors in children and to predict rare adverse events occurrence after vaccination^39–41^. At the best of our knowledge there are no applications of this model to predict CTCAE in GEPTNET patients.

Our analysis suggests that line of PRRT administration, age, gender and ECOG performance status are possible predictors of CTCAE. For several outcomes, these results are confirmed by the internal validation based on subsampling procedure. The predictive performance of our models, assessed by AUC, was quite poor for several outcomes, due to the small sample size and the low number of events observed in our dataset. However, the performance of anaemia, creatinine and eGFR predictive models was good, suggesting the reproducibility of the results for these outcomes.

The FLIC model appears to be a promising method for prediction of CTCAE in the GEPNET management. Approaches similar to the one used in this paper can address clinician decision making, modifying treatment sequencing or suggesting radiodrug reduction activity. However, this study has some major limitations related to the small sample size, the low number of G3-G4 adverse events and the absence of an external validation. Further investigations, including multicentre studies, will be planned to improve the knowledge regarding the predictive factors of CTCAE in GEPNET patients.

## Conclusion

The development of predictive models would be extremely useful to improve GEPNET patient decision making and to avoid PRRT-related adverse events onset. CTCAE occurrence during PRRT treatment can be assessed applying penalised approaches like FLIC model. Subsampling procedure is a useful tool to perform internal validation of the model. External validation is needed before clinical implementation.

## Supplementary information

Supplementary Information.

## Data Availability

The data that support the findings of this study are available from the corresponding author (FS), upon reasonable request.
