# Author Correction: Application of FLIC model to predict adverse events onset in neuroendocrine tumors treated with PRRT

**DOI:** 10.1038/s41598-023-28977-3

**Published:** 2023-01-31

**Authors:** Federica Scalorbi, Giovanni Argiroffi, Michela Baccini, Luca Gherardini, Valentina Fuoco, Natalie Prinzi, Sara Pusceddu, Enrico Matteo Garanzini, Giovanni Centonze, Margarita Kirienko, Ettore Seregni, Massimo Milione, Marco Maccauro

**Affiliations:** 1 Nuclear Medicine Department, Foundation IRCCS, Istituto Nazionale Tumori, Milan, Italy; 2 Department of Statistics, Computer Science, Applications (DiSIA), University of Florence, Florence, Italy; 3 Medical Oncology, Foundation IRCCS, Istituto Nazionale Tumori, Milan, Italy; 4 Diagnostic Imaging Department, Foundation IRCCS, Istituto Nazionale Tumori, Milan, Italy; 5 Pathology Unit, Foundation IRCCS, Istituto Nazionale Tumori, Milan, Italy

Correction to: *Scientific Reports* 10.1038/s41598-021-99048-8, published online 30 September 2021

The original version of this Article contained errors in the spelling of the authors Federica Scalorbi, Giovanni Argiroffi, Michela Baccini, Luca Gherardini, Valentina Fuoco, Natalie Prinzi, Sara Pusceddu, Enrico Matteo Garanzini, Giovanni Centonze, Margarita Kirienko, Ettore Seregni, Massimo Milione & Marco Maccauro, which were incorrectly given as F. Scalorbi, G. Argiroffi, M. Baccini, L. Gherardini, V. Fuoco, N. Prinzi, S. Pusceddu, E. M. Garanzini, G. Centonze, M. Kirienko, E. Seregni, M. Milione & M. Maccauro, respectively.

The original Article has been corrected.

