# Supplementary material for: Application of FLIC model to predict adverse events onset in neuroendocrine tumors treated with PRRT
Source: Sci Rep. 2021 Sep 30;11:19490. doi: 10.1038/s41598-021-99048-8 (PMC8484673; doi:10.1038/s41598-021-99048-8)
Supplement: Supplementary file 1 — Supplementary Information. [file 41598_2021_99048_MOESM1_ESM.pdf]

	Anaemia	Thrombo-cytopenia	Leukopaenia	Neutro-paenia	INR_ increase	AST_ increase	ALT_ increase	GGT_ increase	Creatinina_ increase	EGFR_ increase	Bilirubine_ increase	Albumine_ decrease
Gender	1.889	0.872	0.915	0.167	0.136	0.065	0.170	0.986	2.114	0.563	0.413	0.269
Age	0.020	0.035	0.005	0.007	0.011	0.005	0.006	0.044	0.091	0.175	0.036	0.079
WHO grading	0.081	0.944	0.022	0.005	0.292	0.179	0.125	0.371	0.258	0.460	0.226	0.000
PRRT line	2.658	0.743	0.812	0.783	0.214	0.878	0.033	2.712	0.541	0.265	0.132	0.183
Tumor primary location	0.164	0.047	0.102	0.045	0.352	0.071	0.662	1.104	0.529	1.306	0.244	0.088
ECOG-PS	1.263	0.135	0.061	0.059	0.207	0.037	0.106	0.274	0.333	0.123	0.005	2.247
Chemotherapy	0.192	0.068	0.411	0.015	0.360	0.619	0.759	0.586	0.351	0.295	0.057	0.118
Everolimus	1.468	0.773	0.461	0.231	0.149	0.191	0.082	1.733	1.461	0.708	0.764	0.854
METNET	1.163	0.540	0.106	0.564	1.185	0.199	0.856	0.069	0.056	0.111	0.937	0.071
Splenectomy	0.121	3.050	2.044	0.364	0.343	0.041	1.812	0.809	0.654	0.011	0.097	0.069
N° metastatic sites	0.078	0.035	0.057	0.017	0.037	0.324	0.884	0.042	0.053	0.021	0.067	0.087

Tab S1: mean absolute effects of the covariates arisen from subsampling
